# Association between outdoor air pollution and chronic rhinosinusitis patient reported outcomes

**DOI:** 10.1186/s12940-022-00948-7

**Published:** 2022-12-21

**Authors:** S. Peeters, C. Wang, E. M. Bijnens, D. M. A. Bullens, W. J. Fokkens, C. Bachert, P. W. Hellings, T. S. Nawrot, S. F. Seys

**Affiliations:** 1 Department of Microbiology, Allergy and Clinical Immunology Research Group, Immunology & Transplantation, Herestraat 49/811, 3000 Louvain, KU Belgium; 2 Centre for Environmental Sciences, Hasselt University, Hasselt, Belgium; 3 Clinical Division of Paediatrics, UZ Leuven, Leuven, Belgium; 4 Department of Otorhinolaryngology, Amsterdam University Medical Centres, Location AMC, Amsterdam, The Netherlands; 5 Department of Otorhinolaryngology, Upper Airways Research Laboratory, Ghent University, Ghent, Belgium; 6 Department of Clinical Science, Intervention and Technology, Division of ENT Diseases, Karolinska Institutet, Stockholm, Sweden; 7 Department of Otorhinolaryngology-Head and Neck Surgery, UZ Leuven, Leuven, Belgium; 8 Department of Public Health and Primary Care, Environment and Health Unit, KU Leuven, Leuven, Belgium

**Keywords:** Chronic rhinosinusitis, Outdoor air pollution, Patient reported outcome measures

## Abstract

**Background:**

The aetiology of chronic rhinosinusitis (CRS) is multifactorial with a complex interplay between environmental, microbial endogenous and genetic factors. The impact of outdoor air pollution on prevalence or severity of CRS remains largely unknown.

**Methods:**

Real-life geolocation data (2017–2018, Belgium) from 278 CRS patients (2576 health records) using the mySinusitisCoach mobile application were analysed to calculate the patients’ individual exposure to outdoor air pollutants (ozone (O_3_), black carbon (BC), nitrogen dioxide (NO_2_) and particulate matter with diameter < 2.5 μm (PM_2.5_)) and to associate these pollutants with the patients’ sinus related symptoms measured at multiple occasions by visual analogue scale (VAS).

**Results:**

The adjusted seasonal model for the spring–summer (*n* = 1000 health entries, *N* = 83 patients) population revealed an increase of 6.07 (*p* < 0.0001) in overall CRS symptom scoring for an interquartile range (IQR) increase in exposure to O_3_ (26.9 μg/m^3^). An increase of 1.69 (*p* = 0.05) in total CRS symptom scoring was observed for an IQR increase of PM_2.5_ (7.1 µg/m^3^) exposure. Sex-stratified analysis in the spring–summer population showed significant interaction between air pollution and sex with male patients having higher total CRS symptom scores for an IQR increase in exposure to PM_2.5_ (3.52, *p* = 0.001), and O_3_ (8.33, *p* < 0.0001), while no significant association with symptom severity was seen in the female patients. In the analysis stratified by comorbid asthma, CRS patients with comorbid asthma had higher total CRS symptoms for an IQR increase in exposure to PM_2.5_ (2.58, *p* = 0.04) and O_3_ (7.72, *p* < 0.0001) while the patients without comorbid asthma had no significant symptom increases.

**Conclusion:**

Exposure to outdoor air pollution is associated with increased symptom severity in CRS patients. The extent to which CRS patients are sensitive to outdoor air pollution exposure varies per season and depends on their sex and comorbid asthma status. mHealth technology has the potential to reveal novel insights on the patients’ exposome and disease severity in the real-life situation.

**Supplementary Information:**

The online version contains supplementary material available at 10.1186/s12940-022-00948-7.

## Introduction

Chronic rhinosinusitis (CRS) in adults is defined as an inflammation of the paranasal sinuses and nose with symptoms being present consecutively for more than 12 weeks [1, 2]. It is a common disease, with an overall prevalence of 10.9% in Europe [2, 3]. Two major phenotypes are characterized within CRS, namely CRS with nasal polyps (CRSwNP) and CRS without nasal polyps (CRSsNP) [1, 4]. Depending on the type of inflammation present in the sino-nasal cavities, different endotypes can be distinguished of which type 2 and non-type 2 impact the treatment decisions [5, 6]. The aetiology of CRS is multifactorial, which makes it challenging to identify the exact causal elements of the disease [7]. Genetic factors, environmental factors and the host microbiome have been most commonly reported [1]. Among environmental factors, airway infections, history of smoking, allergies and air pollution have been associated with CRS but their relative contribution to disease severity and progression remains unclear [1].

Outdoor air pollutants are a mixture of harmful components such as ozone (O_3_) and nitrogen dioxide (NO_2_) and particulate matter (PM) with diameter < 2.5 µm (PM_2.5_) and < 10 μm (PM_10_). The association between outdoor air pollution and certain diseases like cardiovascular disease, asthma and chronic obstructive pulmonary disease (COPD) has been extensively studied. Asthma is one of the most prevalent comorbidities in CRSwNP patients with 30%—47% of the CRSwNP patients reporting comorbid asthma [8, 9]. Exposure to outdoor air pollution can cause exacerbations and new-onset of asthma [10–12]. The airway epithelial lining protects our body from pollutants or other environmental intruders [11]. Outdoor air pollution may among others, including occupational agents, negatively affect epithelial barrier function thereby contributing to increased CRS severity [13, 14]. Only a limited number of studies have been performed investigating the relationship between CRS and outdoor air pollution. It has been demonstrated that a positive association exists between outdoor air pollution exposure and the prevalence of CRS [15, 16]. Mady et al*.* demonstrated that for each unit increase in PM_2.5_ there was a 1.89 fold increased risk in the proportion of CRSsNP patients who needed sinus surgery, and that exposure to black carbon (BC) was associated with a more pronounced disease presentation in CRSsNP patients [17]. Although scientific evidence on the association between CRS and outdoor air pollution is limited, it appears that long-term exposure to ambient pollutants is related to a higher prevalence of CRS and more severe CRS symptoms. On the other hand, when looking at studies investigating acute effects of highly concentrated pollutants, studies in the context of the World Trade Centre disaster are of particular interest. One 10 year follow up study in firefighters showed a 2-times increased risk of CRS in high compared to low exposed individuals [18].

In this study the association between short-term exposure to outdoor air pollution and CRS symptom severity is investigated using mixed models with real-life data generated by a mobile application used by CRS patients. The availability of longitudinal data and geolocation records enabled us to perform an analysis of CRS symptom severity associated with air pollution exposure. We hypothesized that increased exposure to outdoor air pollution may be associated with an increase in total symptoms that CRS patients experience. This relationship may be modified by sex, age, smoking status, surgery status, presence of nasal polyps and comorbid diseases like COPD, asthma and allergic rhinitis (AR).

## Methods

### Data collection

Real-life data from patients with CRS were collected through a mobile health application called mySinusitisCoach [19]. Patients were considered for use of mySinusitisCoach when they have filled out that they have two or more sino-nasal symptoms and/or have a doctor-based diagnosis of CRS [20]. Patients using the application were asked to fill out a health diary in which they were asked to mark their total CRS symptom severity based on a visual analogue scale (VAS) from 0 (no symptoms) to 100 (maximum symptoms). In addition to the symptom record, the timestamp and geolocation of each entry was recorded. Patients were advised to complete the health diary on a weekly basis. For this study, only entries made in 2017–2018 by patients located in Belgium at time of registration were included. Minors were excluded. The study was approved by the institutional review board of UZ Leuven, Belgium.

### Study population

**Fig. 1:**
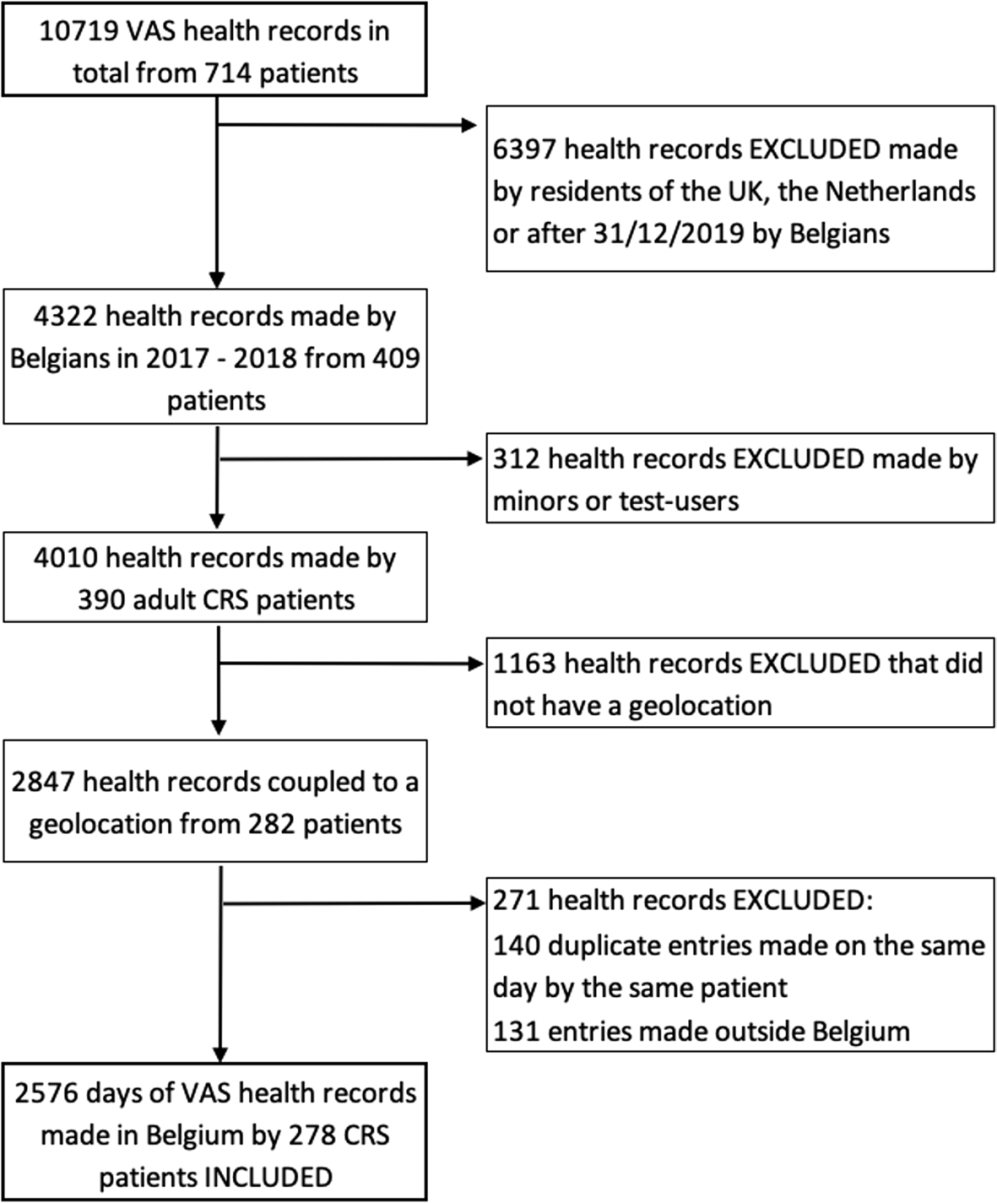
Flowchart of the visual analogue scale (VAS) health record selection process. The final study population consisted of 278 CRS patients above the age of 18 that made 2576 days of VAS health records in Belgium

The database consisted of 10,719 days of unique health records that are available from 714 different CRS patients from the UK, Belgium and the Netherlands. All health records were created between 09/11/2017 and 14/07/2019. After applying exclusion criteria, the final study population consisted of 278 different CRS patients residing in Belgium above the age of 18, with 2,576 corresponding days of health records coupled to a geolocation made in Belgium between 10/11/2017 and 31/12/2018 (Fig. 1). In order to take into account the consistence of the actual exposure and outdoor pollutant concentrations, the population was split up into two seasonal populations: a spring–summer population consisting of health entries made during the months April—September of 2018 (*n* = 1144, patients = 116) and a fall-winter population that consisted of health entries made during the months November and December of 2017 and January – March and October—December of 2018 (*n* = 1432, patients = 222).

### Calculation of individual exposure data and descriptive analysis

The geolocation information was used to estimate the average daily concentrations of O_3_ by taking the 8-h average, and PM_2.5_, BC and NO_2_ by taking the 24-h average. For every health entry, the daily average concentrations were calculated for the day of the entry (lag 0), as well as 1–7 days prior to the entry (lag 1–7) in order to study the lagged effect of the exposures on the recorded symptoms. Daily air pollution levels were modelled by the RIO detrended kriging interpolation model (4 by 4 km) that uses land cover data obtained from satellite images [21]. The RIO model outperforms standard interpolation techniques such as the Ordinary Kriging model as the bias, root mean square error and mean absolute error improve for each O_3_, NO_2_ and PM_10_ [21]. The validation statistics of this interpolation model give an explained spatiotemporal variance of more than 0.74 for BC, 0.78 for NO_2_ and 0.80 for PM_2.5_ [22]. Average daily temperatures and relative humidity recorded in Uccle, Belgium for the respective period were requested at the Royal Meteorological Institute. Distance between individuals’ location and monitors of temperature and humidity was 46.1 (27.9 – 71.5) km (median with interquartile range). Average exposures of each pollutant and relative humidity and temperature were calculated for seven different lag intervals: lag 0–1 (AVG 1), lag 0–2 (AVG 2), lag 0–3 (AVG 3), lag 0–4 (AVG 4), lag 0–5 (AVG 5), lag 0–6 (AVG 6) and lag 0–7 (AVG 7). The production of O_3_ is catalyzed by sunlight and O_3_ concentration displays a strong seasonal pattern which is highest during the spring and summer in Belgium [23]. Therefore, O_3_ exposure was only studied for the spring–summer population, which had relevant UV-levels and O_3_ concentrations. Descriptive statistics of the exposure data were analyzed using Prism Graphpad 6 and R 3.6.3. Spearman’s rank correlations were calculated between the different pollutants on the same day as well as between adjacent days for the same pollutant. Comparisons were made between both seasonal sub-populations for the pollutants and total symptom scoring using the Mann–Whitney U test.

### Statistical analysis

The statistical workflow can be observed in supplementary Figure S1. To evaluate the association between the symptoms and the exposures, linear mixed models were fitted to account for the intercorrelation between each patient’s multiple health entries. Whether to include random intercepts or random slopes was decided based on the intraclass correlation coefficients, which were calculated for the models to assess for the necessary random effects. This was followed by regressing the symptoms on the mean exposure of each pollutant of each of the seven exposure intervals, separately. The Akaike Information Criterion (AIC) was calculated for each model to select the models with the best fitting exposure interval [24]. Possible confounding was considered by creating adjusted models, with the following covariates: outdoor temperature, humidity, sex, age, smoking history, sinus surgery history, nasal polyps and comorbidities such as COPD, AR and asthma, which were selected a priori based on previous literature studying the potential association between air pollution exposure and CRS. The non-numerical covariates were coded as follows; sex: male or female; smoking status: smoker or never smoker; nasal polyp status: CRSsNP or CRSwNP; sinus surgery history, COPD, AR: yes or no; and asthma: asthma since adulthood or asthma since childhood or no. Effect modification of the association between the exposures and the overall CRS symptoms was assessed for these covariates by testing the interaction terms between the exposure and the covariates. Stratified analyses were performed based on the significant effect modifications in the spring–summer population for lag exposure interval AVG 7. For the stratification of asthma, no distinction between ‘asthma since adulthood’ and ‘asthma since childhood’ was made. It was considered that pollution exposure, temperature and relative humidity might have a non-linear effect by fitting models with high-order terms. To avoid multicollinearity temperature and relative humidity were scaled. The AIC was calculated for each model to determine the best fitting order [24]. In the adjusted models the variance inflation factor (VIF) was calculated for each selected adjusted model to check for multicollinearity among the covariates. A VIF of 5 was taken as the cut-off value for the presence of a multicollinearity problem. All mixed models were fitted using the lme4 package in R 3.6.3 [25].

## Results

### Study population

**Fig. 2:**
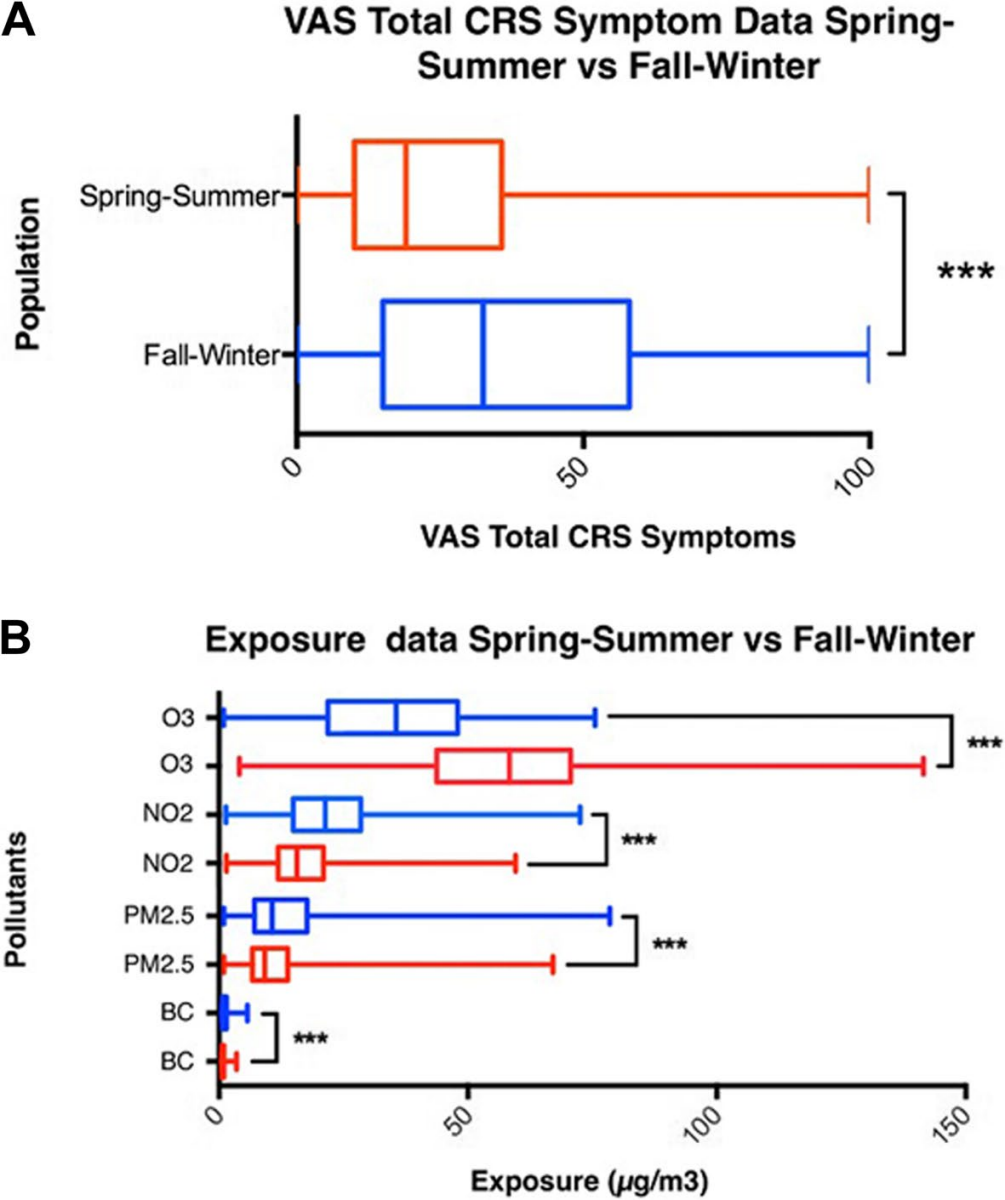
VAS total CRS symptom data and exposure data from both seasonal populations. VAS total CRS symptom data and exposure data presented as boxplots. **A** Distribution of the VAS total CRS symptoms of the spring–summer (red) population (patients = 116, health records = 1144) and the fall-winter (blue) population (patients = 222, health records = 1432). **B** Exposure data generated for the spring–summer (red) population (patients = 116, health records = 1144, exposure days = 9152) and for the fall-winter (blue) population (patients = 222, health records = 1432, exposure days = 11,456) for O_3_, PM_2.5_, BC and NO_2_. *** = *p*-value < 0.001

The characteristics of the study population are shown in Table 1. The average age of the total study population was 44 (± 13.56) years. Man (51.43%) and women (48.56%) were equally represented. 53.02% of the population had nasal polyps and 54.68% of the population had a history of sinus surgery. 49.05% of the population had AR, 6.77% had COPD and 23.02% had asthma (Table 1). The patients had significantly higher total CRS symptoms in the fall and winter compared to the spring and summer (*p* < 0.001) (Fig. 2A, S2). Months with known birch (April) and grass (June-July) pollen season peaks for Belgium did not show increased total CRS symptoms (Figure S2 and consulted at: https://airallergy.sciensano.be).

**Table 1:** Information collected on the study population (*N* = 278)

Characteristics	Total (%)	Total (%)	Total (%)
**Population**	Complete population (*N* = 278)	Spring—Summer population (*N* = 116)	Fall—Winter population (*N* = 222)
**Age, years** *(mean* ± *SD)*	44 ± 13.56	45 ± 13.57	44 ± 13.74
**Sex** *(Male/Female)*	143/135	64/52	100/112
**Nasal polyps**			
CRSwNP	123 (53.02%)	60 (51.72%)	96 (43.24%)
CRSsNP	109 (46.98%)	38 (32.76%)	89 (40.01%)
**History of sinus surgery**	152 (54.68%)	71 (61.21%)	119 (53.60%)
**Smoking status**			
Never smoker	192 (72.18%)	63 (54.31%)	129 (58.11%)
Ex-smoker	62 (23.31%)	28 (24.14%)	48 (21.62%)
Current smoker	12 (4.51%)	4 (3.45%)	9 (4.05%)
**Allergic rhinitis**	129 (49.05%)	48 (41.38%)	103 (46.40%)
**Asthma**			
Childhood onset	15 (6.28%)	8 (6.90%)	14 (6.31%)
Adulthood onset	40 (16.74%)	22 (18.97%)	30 (13.51%)
**COPD**	17 (6.77%)	8 (6.90%)	12 (5.41%)

Age was represented in mean ± standard deviation. 46 patients were missing nasal polyp status, 12 missing smoking status, 15 missing allergic rhinitis status, 39 missing asthma, and 27 missing COPD. It should be noted that when patients made entries in both the spring—summer and in the fall—winter period they were included in both populations

### Outdoor air pollution exposure

As expected, significant pairwise correlations were found for all of the pollutants (Supplementary Table S1, S2).

When comparing the exposures of the spring–summer and the fall-winter population with each other, the O_3_ concentration appeared to be significantly higher in the spring–summer months than in the fall-winter months (*p* < 0.001). BC, PM_2.5_, and NO_2_ concentrations were significantly higher in the fall-winter months compared to the spring–summer months (*p* < 0.001) (Fig. 2B).

### Effect-modification

Sex, age, nasal polyp status, COPD, adulthood onset asthma and history of sinus surgery were found to be effect-modifiers in the spring–summer population (Table 2). Sex was found to be an effect-modifier of the association between the total CRS symptom score and BC, PM_2.5_, and NO_2_ exposure, as male patients had a greater change in the total CRS symptom scoring per 1-unit increment in the pollutants, compared with female patients. Comorbid COPD and adulthood onset asthma were found to be effect-modifiers of the association between O_3_ exposure and the total CRS symptom scoring, with a greater change in the symptom scoring of COPD or adulthood onset asthma patients per 1-unit increment in the pollutants. Patients with a history of sinus surgery showed a smaller change in the total CRS symptom score per 1-unit increment in BC, PM_2.5_, and NO_2_ exposure compared to patients without a history of sinus surgery. Comorbid AR, smoking status and childhood onset asthma did not modify any of the associations.

**Table 2:** Effect-modification observed for the spring–summer CRS population

Spring–Summer Population	BC		PM_2.5_		NO_2_		O_3_	
	Estimate	*p*-value	Estimate	*p*-value	Estimate	*p*-value	Estimate	*p*-value
Sex (Male)	12.1	0.00495**	0.551	0.011*	0.539	0.0269*	0.0997	0.146
Age (+ 1 year)	-0,0353	0.820	0.00159	0.843	-0.00352	0.686	0.0101	2.18E-4***
CRSwNP	-6.41	0.206	-0.106	0.686	-0.540	0.0402*	0.00686	0.934
Comorbid AR	-2.63	0.55	0.177	0.145	-0.103	0.675	0.0651	0.376
Comorbid adulthood onset asthma	-1.65	0.760	0.544	0.0999	-0.425	0.141	0.344	3.65E-05***
Comorbid childhood onset asthma	1.20	0.801	0.146	0.550	0.0752	0.772	0.126	0.107
Comorbid COPD	13.0	0.0664	0.461	0.150	0.443	0.220	0.432	5.91E-06***
Smoker	-1.35	0.810	0.0174	0.946	-0.0791	0.807	0.156	0.0663
Prior Sinus Surgery	-12.4	0.00388**	-0.806	9.85E-04***	-0.690	0.0023**	0.129	0.0754

Effect modification displayed for the association between the total CRS symptom score covariates and a 1-unit (1 µg/m3) increment of the pollutants

^*^ *p*-value < 0.05

^**^ p-value < 0.01

^***^ *p*-value < 0.001

### Associations between symptom severity and exposure to pollutants using repeated measures

**Fig. 3:**
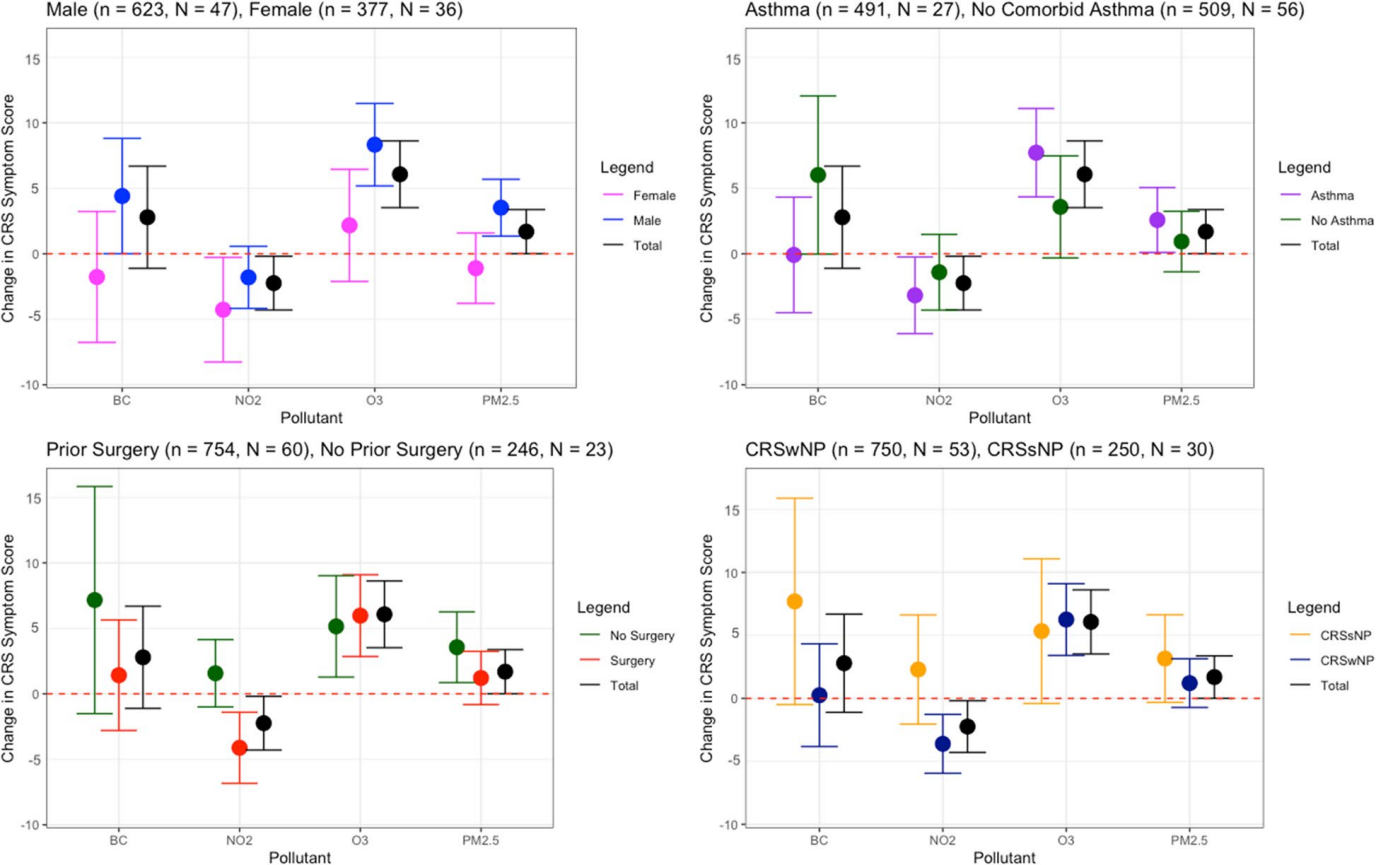
Relationship between CRS symptom severity and outdoor air pollution for the spring–summer population. Change in total CRS symptom scoring for the total spring–summer population (n = 1000 health entries, N = 83 patients) as perceived by the patients after being exposed to an interquartile range (IQR) increase of the pollutant the week before the entry (lag 0 – lag 7). IQR BC = 0.5 µg/m^3^, IQR NO_2_ = 9.1 µg/m^3^, IQR O_3_ = 26.9 µg/m^3^, IQR PM_2.5_ = 7.1 µg/m^3^. The population was adjusted for outdoor temperature, humidity, sex, age, past smoking status, sinus surgery history, nasal polyp status and comorbidities: COPD, AR and asthma. **A** Stratification based on sex. **B** Stratification based on comorbid asthma status. **C** Stratification based on sinus surgery history. **D** Stratification based on nasal polyp status

In the adjusted models for the spring–summer population (*n* = 1000 health entries, *N* = 83 patients), each patient made an average of 12 health entries in mySinusitisCoach. The largest lag interval (AVG 7) was selected for all pollutants as the best fitting exposure term. The models with temperature, humidity, and pollution exposure as first order terms were selected as best fitting models (Table S3). A significant increase of 6.07 (*p* < 0.0001) in total CRS symptom score was found for an IQR increase in the average O_3_ (26.9 µg/m^3^) exposure the week before the entry (Fig. 3). A significant decrease of 2.24 (*p* = 0.03) in total CRS symptom score was found for an IQR increase in NO_2_ (9.1 µg/m^3^) exposure. An increase of 1.69 (*p* = 0.05) in total CRS symptom scoring was observed for an IQR increase of PM_2.5_ (7.1 µg/m^3^) exposure. No significant changes in symptoms were observed for BC (Fig. 3). In the adjusted models for the fall-winter population (*n* = 1149 health entries, *N* = 153 patients) no significant changes in total CRS symptom scores were observed (Supplementary Figure S4B).

#### Sex

Exposure to O_3_ and PM_2.5_ caused a significant increase in total CRS symptom scoring in males, with the observed changes for an IQR increase being 3.52 (*p* = 0.0153) for PM_2.5_, and 8.33 (*p* < 0.0001) for O_3_ (Fig. 3A) In females, significant change was only detected with NO_2_ where an IQR increase in NO_2_ was associated with 4.27 (*p* = 0.03) decrease in symptom score (Fig. 3A).

#### Asthma

CRS patients with comorbid asthma showed increased total CRS symptom scores with increased exposures, where the association with an IQR increase in PM_2.5_ was 2.58 (*p* = 0.04) and in O_3_ was 7.72 (*p* < 0.0001) and had less symptoms for an IQR increase in NO_2_ (-3.17, *p* = 0.03) (Fig. 3B). No significant estimates of symptom scores were seen in CRS patients without asthma.

#### Prior sinus surgery status

Prior sinus surgery status did affect the association between the pollutants and the total CRS symptoms. A significant change in CRS total symptom score was observed after exposure to NO_2_ (-4.12 per IQR increase, *p* = 0.0300) and O_3_ (5.97 per IQR increase, *p* = 1.96E-04) in the CRS population that had prior sinus surgery. For the CRS patients without history of sinus surgery an IQR increase of exposure of PM_2.5_ and O_3_ was associated with an increase of 3.56 (*p* = 0.0104), and 5.15 (*p* = 0.00965) respectively (Fig. 3C).

#### CRS phenotype

A significant association was found for NO_2_ as well as O_3_ exposure and the total CRS symptom scoring of the CRSwNP patients. A change of -3.61 per IQR increase (*p* = 0.002) for NO_2_ and of 6.25 per IQR increase (*p* < 0.0001) for O_3_ was observed, whereas the CRSsNP population did not show significant total CRS symptom associations to any of the pollutants (Fig. 3D).

## Discussion

Although exposure to environmental and occupational factors such as tobacco smoke, fire smoke and dust exposure have been linked with the prevalence of CRS, the association between the prevalence and severity of CRS and outdoor air pollution is far less understood [1, 4, 26]. According to a recent systematic review, 10 relevant manuscripts have been published before demonstrating higher odds of CRS particularly with PM exposure [27]. To our knowledge this is the first study using mixed models to describe symptom severity data of CRS patients in relation to outdoor air pollution exposure. We here demonstrated that exposure to O_3_ and PM_2.5_ leads to an increased CRS symptom scoring in CRS patients during the spring–summer period. Sensitivity of CRS patients to outdoor air pollution exposure depends on their sex, presence of comorbid asthma and history of sinus surgery.

In our study, we speculated that the models created for the spring–summer population were better in reflecting personal exposure to outdoor air pollution than the models fitted for the fall-winter population. It has been previously demonstrated that during colder months people spend more time inside and aerate their households less [28]. Therefore, a stronger association has been observed between indoor exposure and outdoor exposure during warmer months compared to the colder months [28]. It was observed that in the fall-winter population CRS symptom scores were higher than those of the spring–summer population, while the association between outdoor air pollution and increased CRS symptoms were more present in the spring–summer population. This is likely due to the fact that during the winter viral infections are more prevalent among the population which often exacerbate CRS symptoms [29]. This may thus overshadow the effects of the pollutants on the symptoms, which together with the fact that the fall-winter population models contain less accurate estimates of the exposure, explain the observation of nonsignificant changes in the total CRS symptoms after exposure in this population.

The unstratified adjusted spring–summer population showed a significant increase and decrease in symptom score for O_3_ and NO_2_ exposure respectively. The increase of symptoms associated with O_3_ exposure is approximately three times bigger than the decrease of symptoms associated with NO_2_ exposure when comparing their IQR estimates. O_3_ exposure has previously been shown to cause damage to the nasal epithelium and to cause increased nose and sinus symptoms, such as rhinorrhea, nasal dryness, nasal obstruction, epistaxis and olfactory impairment, in non-CRS patients [30–32]. The observed decrease in symptoms associated with NO_2_ exposure could be explained by the interaction between O_3_ and NO_2_. O_3_ is formed by a photochemical reaction requiring NO_x_ and volatile organic compounds and an inverse relationship exists between O_3_ and NO_2_ [33, 34].

No significant effects were observed for black carbon. Mady et al. observed more pronounced disease progression in CRS patients after BC exposure [17]. The relative low sample size may have been the reason why we failed to detect the association between BC and the symptoms in the adjusted spring–summer population.

In our population, male patients are more sensitive to O_3_ and PM_2.5_ exposure compared to female patients. In literature, evidence of effect modification by sex on respiratory health remains uncertain as studies most often find stronger effects of pollution exposure among women, however certain studies have also suggested stronger effects among men [35]. Confounding may exist because of unmeasured characteristics, including biological factors related to deposition, reactivity, and hormonal influences on chemical transport and systemic regulation as well as gender-related explanations such as exposures to indoor allergens and cleaning agents, job-related chemical exposures, and differing exposure and response to psychosocial stressors [35].

A remarkable finding of this study is that CRS patients with comorbid asthma are sensitive to outdoor air pollution while non-asthmatic CRS patients are not. This could be explained by the fact that asthma exacerbations are known to be associated with outdoor air pollution and that poor asthma control has a negative effect on CRS patient outcomes [11, 36, 37]. Self-reported AR did not affect the relationship between air pollutants and symptoms in CRS patients. Increased total CRS symptoms over the year also did not track with tree or grass pollen seasons. This is not surprising since the prevalence of AR is not higher in CRS compared to the general population except for specific phenotypes like allergic fungal rhinosinusitis or central compartment atopic disease [38]. Also, local polyclonal IgE is produced in the upper airways of CRS patients irrespective of their allergy status [39]. It has been shown that superantigens such as Staphylococcus Enterotoxin B are drivers of this polyclonal IgE response [40].

Sinus surgery significantly changes the airflow and increases the deposition of particles in the sinuses, possibly making patients more sensitive after surgery [41]. However, we observed significant symptom increases after exposure to PM_2.5_ in the group that did not have prior sinus surgery and no significant symptom changes in the prior surgery group. This may be due to the fact that the time between the last sinus surgery and symptom reporting varies between patients and no information was available on patient’s date of last sinus surgery. For O_3_ we did not observe an impact of prior sinus surgery.

A previous study demonstrated that CRSsNP patients are more sensitive to PM and BC compared to CRSwNP patients [17]. In our population the observed increases for the CRSsNP patients for BC and PM_2.5_ were not significant, however changes in symptom severity were higher for these pollutants in CRSsNP compared to CRSwNP patients. 

Lastly, smoking status did not affect the relationship between air pollutants and symptoms in CRS patients in this study. Exposure to cigarette smoke on itself, either active or passive, though contributes to chronic rhinosinusitis according to a review by Reh et al. [42].

Certain limitations of this study should be noted. A first limitation of the study relates to the fact that we calculated exposure data for the coordinates recorded on the day of entry and assumed the same location for up to the prior seven days. Besides, outdoor pollutant concentration does not necessarily reflect the actual personal exposure of the patients to pollutant since indoor pollutant concentrations have been shown to impact patients’ health variables as well [28]. Secondly, patients’ self-reported outcome measures were based on total sinusitis symptoms in this analysis. In future studies with larger numbers of patients reporting on specific symptoms of CRS, it would be interesting to study how specific symptoms such as impaired smell, facial pain and nasal blockage are affected in CRS patients by air pollution. This type of analysis featuring longitudinal real-life data can have many significant implications in further understanding the real-world impacts of pollution on CRS symptom outcomes. Therefore, we suggest a follow-up study to be performed with a larger dataset on a larger geographical scale.

In conclusion, several novel findings have been observed in the present study for CRS patient symptomatology in relationship to air pollution exposure. During the spring and summer period in Belgium, an association between the total CRS symptoms and O_3_ as well as PM_2.5_ exposure was demonstrated in CRS patients. Additionally, male patients and patients with comorbid asthma appeared to be more sensitive to the exposure of several of the pollutants compared to the female CRS patients and patients without comorbid asthma.

## Supplementary Information

**Additional file 1:** **Figure S1.** Workflow depicting hierarchical model build-up and selection process. **Figure S2.** Variation in VAS of the total CRS symptoms per month. **Figure S3. **Relationship between CRS symptom severity and outdoor air pollution for the spring-summer population. **Figure S4. **Relationship between CRS symptom severity and outdoor air pollution for the fall - winter population. **Table S1.** Spearman’s rank correlation for same pollutants on adjacent lag days. **Table S2.** Spearman’s rank correlation for different pollutants on lag day 0. **Table S3.** AIC for model selection of the adjusted spring-summer population. 

## Data Availability

Not applicable.

## Abbreviations

AIC: Akaike Information Criterion
AR: Allergic Rhinitis
BC: Black Carbon
COPD: Chronic Obstructive Pulmonary Disease
CRS: Chronic Rhinosinustis
CRSsNP: Chronic Rhinosinustis without Nasal Polyps
CRSwNP: Chronic Rhinosinustis with Nasal Polyps
IQR: Interquartile Range
NO_2_: Nitrogen Dioxide
O_3_: Ozone
PM: Particulate Matter
PM_10_: Particulate Matter < 10 µm
PM_2.5_: Particulate Matter < 2.5 µm
VAS: Visual Analoge Scale
VIF: Variance Inflation Factor
