# Supplementary material for: Association between outdoor air pollution and chronic rhinosinusitis patient reported outcomes
Source: Environ Health. 2022 Dec 21;21:134. doi: 10.1186/s12940-022-00948-7 (PMC9769041; doi:10.1186/s12940-022-00948-7)
Supplement: Supplementary file 1 — Additional file 1: Figure S1. Workflow depicting hierarchical model build-up and selection process. Figure S2. Variation in VAS of the total CRS symptoms per month. Figure S3. Relationship between CRS symptom severity and outdoor air pollution for the spring-summer population. Figure S4. Relationship between CRS symptom severity and outdoor air pollution for the fall - winter population. Table S1. Spearman’s rank correlation for same pollutants on adjacent lag days. Table S2. Spearman’s rank correlation for different pollutants on lag day 0. Table S3. AIC for model selection of the adjusted spring-summer population. [file 12940_2022_948_MOESM1_ESM.docx]

**ONLINE SUPPLEMENT**

**Supplementary figures**

**
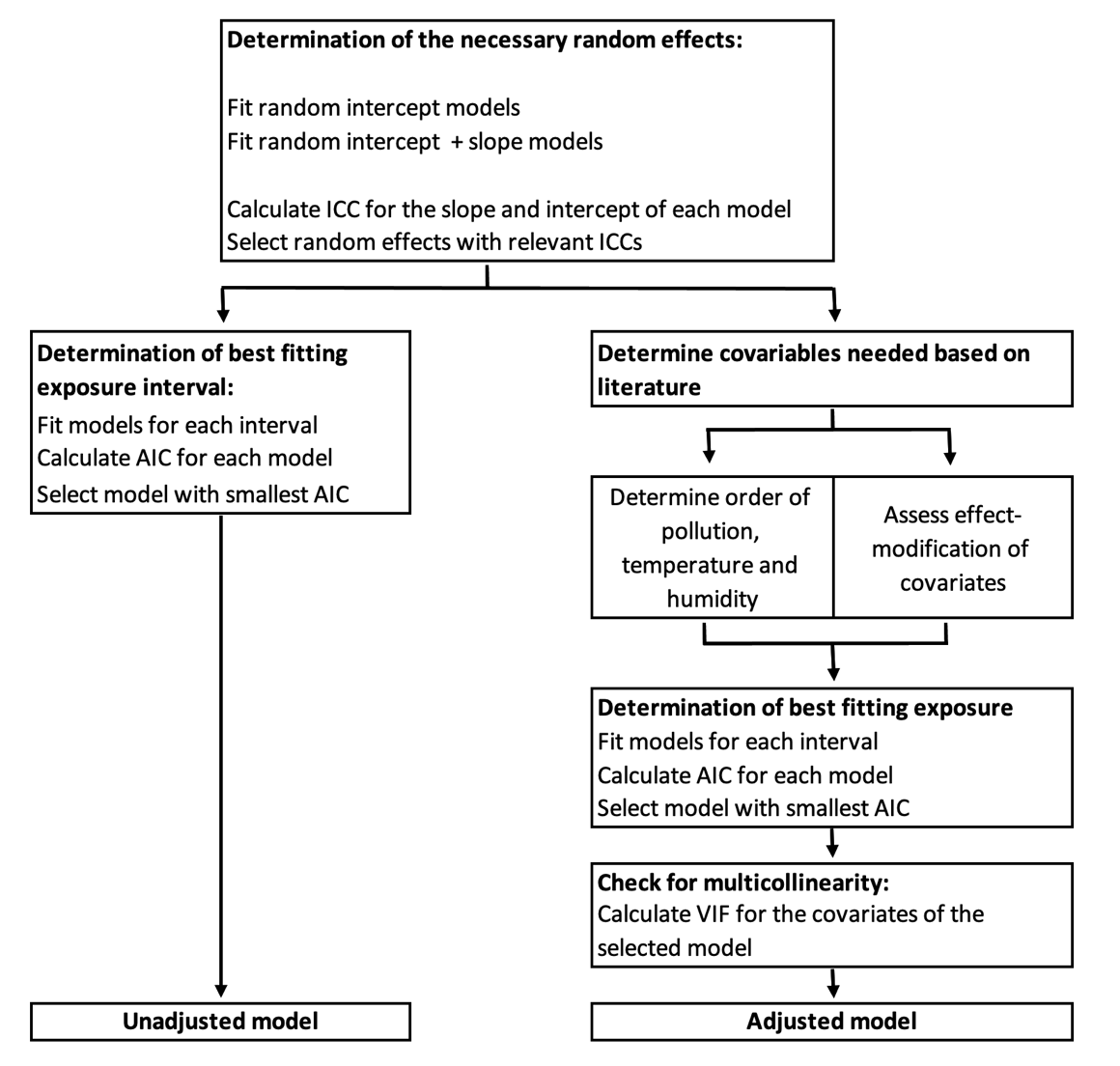
**

**B.**

**A.**

Figure S1. Workflow depicting hierarchical model build-up and selection process.

**A**. For the unadjusted models per pollutant according to the seven average exposure intervals (AVG 1 – 7), seven random intercept models and seven random slope models were fitted for each BC, PM_2.5_, and NO_2_ for the spring-summer population consisting of health entries made during the months April – September (spring-summer population, n = 1144, patients = 116) and the fall-winter population consisting of health entries made during the months January- March and October - December (n = 1432, patients= 222). For O_3_ seven random intercept models and seven random slope models were fitted for the spring-summer population for exposure intervals AVG 1 – 7. **B.** The adjusted models were created including covariates: outdoor temperature, humidity, sex, age, past smoking status, sinus surgery history, nasal polyp status and comorbidities such as COPD, AR and asthma. Temperature and humidity were scaled before being integrated in the models. For PM_2.5_, NO_2_ random intercept models were fitted and for BC random coefficient models were fitted for exposure intervals AVG 1 – 7 for the spring-summer population (n = 1000, patients = 83) and the fall-winter population (n = 1149, patients = 153). For O_3_ a random intercept model was fitted for the spring-summer population for exposure intervals AVG 1 – 7. ICC: intraclass correlation coefficient, AIC: Akaike’s information criterion, VIF: variance inflation factor.

**
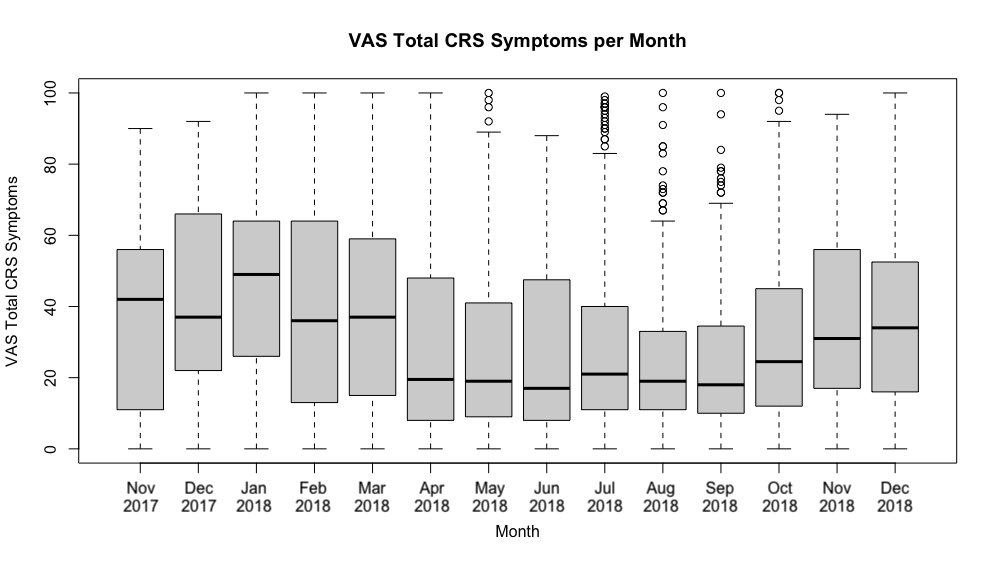
**

**Figure S2. Variation in VAS of the total CRS symptoms per month.**

Variation in the visual analogue scale (VAS) scoring of the total CRS symptoms as experienced by the patients for all studied months. Boxplots display the median and the quartiles.

**Change in Total CRS Symptoms per IQR Pollutant Increase for the Unadjusted Spring-Summer Population**


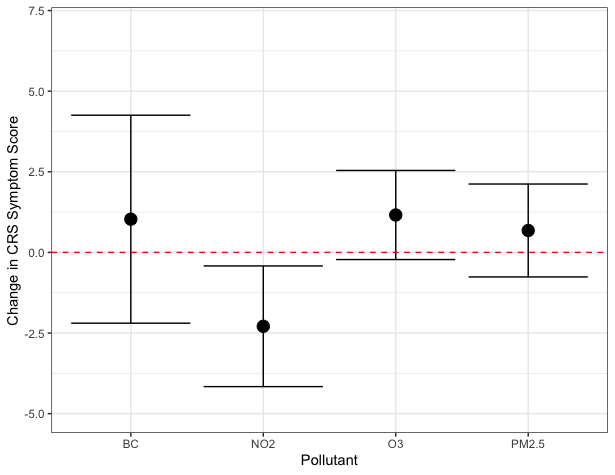


**Figure S3. Relationship between CRS symptom severity and outdoor air pollution for the spring-summer population.**

Change in total CRS symptom scoring as perceived by the patient after being exposed to an interquartile range (IQR) increase of the pollutant of the pollutant the week before the entry (lag 0 – lag 7) for the unadjusted spring summer population (n = 1144 health entries, N = 116 patients).

**Change in Total CRS Symptoms per IQR Pollutant Increase for the Fall-Winter Population**

**A.**

**B.**


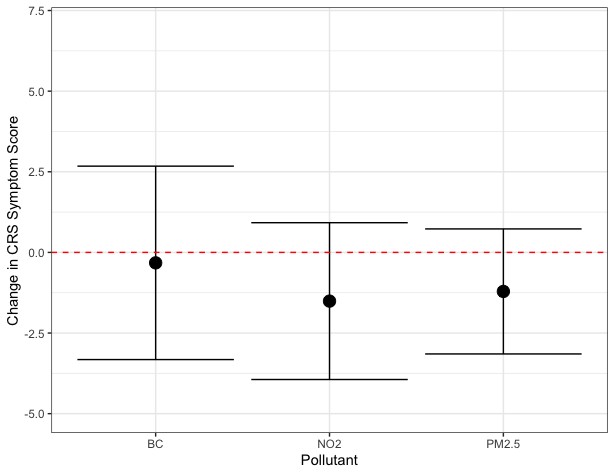

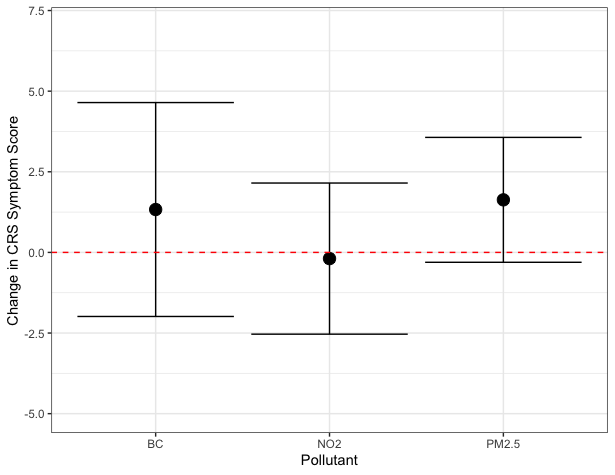


**Figure S4. Relationship between CRS symptom severity and outdoor air pollution for the fall - winter population.**

Change in total CRS symptom scoring as perceived by the patient after being exposed to an interquartile range (IQR) increase of the pollutant of the pollutant the week before the entry (lag 0 – lag 7). **A.** Unadjusted mixed models (n = 1433 health entries, N= 222 patients). **B.** Adjusted mixed models (n = 1149 health entries, N = 153 patients), the population was adjusted for outdoor temperature, humidity, sex, age, past smoking status, sinus surgery history, nasal polyp status and comorbidities: COPD, AR and asthma. IQR: interquartile range.

|  | **BC** | | **PM_2.5_** | | **NO_2_** | | **O_3_** | |
| --- | --- | --- | --- | --- | --- | --- | --- | --- |
|  | rho | p-value | rho | p-value | rho | p-value | rho | p-value |
| **lag 0 - lag 1** | 0.622 | <2.2E-16*** | 0.656 | <2.2E-16*** | 0.628 | <2.2E-16*** | 0.807 | <2.2E-16*** |
| **lag 1 - lag 2** | 0.611 | <2.2E-16*** | 0.644 | <2.2E-16*** | 0.622 | <2.2E-16*** | 0.795 | <2.2E-16*** |
| **lag 2- lag 3** | 0.618 | <2.2E-16*** | 0.624 | <2.2E-16*** | 0.634 | <2.2E-16*** | 0.804 | <2.2E-16*** |
| **lag 3 - lag 4** | 0.595 | <2.2E-16*** | 0.637 | <2.2E-16*** | 0.599 | <2.2E-16*** | 0.800 | <2.2E-16*** |
| **lag 4 - lag 5** | 0.595 | <2.2E-16*** | 0.638 | <2.2E-16*** | 0.600 | <2.2E-16*** | 0.806 | <2.2E-16*** |
| **lag 5 - lag 6** | 0.587 | <2.2E-16*** | 0.653 | <2.2E-16*** | 0.592 | <2.2E-16*** | 0.793 | <2.2E-16*** |
| **lag 6 - lag 7** | 0.630 | <2.2E-16*** | 0.650 | <2.2E-16*** | 0.641 | <2.2E-16*** | 0.817 | <2.2E-16*** |

Table S1. Spearman’s rank correlation for same pollutants on adjacent lag days.

Presence of correlation between the concentrations of the same pollutants on adjacent days. ***: p<0.001.

|  | **BC** | | **PM_2.5_** | | **NO_2_** | |
| --- | --- | --- | --- | --- | --- | --- |
|  | rho | p-value | rho | p-value | rho | p-value |
| **PM_2.5_** | 0.687 | <2.2E-16*** |  |  |  |  |
| **NO_2_** | 0.89 | <2.2E-16*** | 0.585 | <2.2E-16*** |  |  |
| **O_3_** | -0.551 | <2.2E-16*** | -0.294 | <2.2E-16*** | -0.589 | <2.2E-16*** |

Table S2. Spearman’s rank correlation for different pollutants on lag day 0.

Presence of correlation between the different pollutants their concentrations on the same day. ***: p<0.001.

|  |  | **AIC** | | | |
| --- | --- | --- | --- | --- | --- |
|  |  | **First degree temperature, humidity, pollutant** | | | |
|  |  | **BC** | **NO_2_** | **PM_2.5_** | **O_3_** |
| **Exposure interval** | **AVG1** | 8424.688 | 8395.283 | 8396.593 | 8388.701 |
|  | **AVG2** | 8422.851 | 8393.14 | 8396.222 | 8383.598 |
|  | **AVG3** | 8426.295 | 8392.026 | 8395.419 | 8383.209 |
|  | **AVG4** | 8422.14 | 8390.719 | 8394.152 | 8380.124 |
|  | **AVG5** | 8412.978 | 8390.099 | 8392.856 | 8375.425 |
|  | **AVG6** | 8405.411 | 8389.354 | 8390.316 | 8373.525 |
|  | **AVG7** | 8387.266 | 8387.996 | 8388.618 | 8372.755 |
|  |  | **Second degree temperature, First degree humidity, pollutant** | | | |
|  |  | **BC** | **NO_2_** | **PM_2.5_** | **O_3_** |
|  | **AVG1** | 8430.947 | 8395.283 | 8404.217 | 8394.244 |
|  | **AVG2** | 8429.099 | 8393.14 | 8403.717 | 8387.675 |
|  | **AVG3** | 8431.076 | 8392.026 | 8402.882 | 8385.098 |
|  | **AVG4** | 8417.355 | 8390.719 | 8408.79 | 8380.92 |
|  | **AVG5** | 8420.581 | 8390.099 | 8400.26 | 8376.156 |
|  | **AVG6** | 8411.672 | 8389.354 | 8397.527 | 8376.628 |
|  | **AVG7** | 8402.033 | 8395.269 | 8395.603 | 8375.868 |
|  |  | **Second degree humidity, First degree humidity, pollutant** | | | |
|  |  | **BC** | **NO_2_** | **PM_2.5_** | **O_3_** |
|  | **AVG1** | 8430.947 | 8395.283 | 8408.047 | 8399.654 |
|  | **AVG2** | 8414.809 | 8393.14 | 8407.684 | 8394.808 |
|  | **AVG3** | 8439.766 | 8392.026 | 8406.411 | 8394.171 |
|  | **AVG4** | 8439.085 | 8390.719 | 8412.189 | 8390.536 |
|  | **AVG5** | 8427.403 | 8390.099 | 8403.177 | 8385.852 |
|  | **AVG6** | 8402.465 | 8389.354 | 8399.412 | 8383.924 |
|  | **AVG7** | 8402.794 | 8394.628 | 8396.327 | 8382.941 |
|  |  | **Second degree pollutant, First degree temperature, humidity** | | | |
|  |  | **BC** | **NO_2_** | **PM_2.5_** | **O_3_** |
|  | **AVG1** | 8425.144 | 8405.697 | 8408.047 | 8402.557 |
|  | **AVG2** | 8418.948 | 8403 | 8404.807 | 8396.687 |
|  | **AVG3** | 8426.118 | 8400.96 | 8404.603 | 8396.328 |
|  | **AVG4** | 8422.771 | 8390.719 | 8412.189 | 8393.365 |
|  | **AVG5** | 8411.149 | 8399 | 8399.534 | 8387.709 |
|  | **AVG6** | 8401.514 | 8398.25 | 8397.283 | 8383.296 |
|  | **AVG7** | 8394.187 | 8396.823 | 8396.186 | 8380.85 |

Table S3. AIC for model selection of the adjusted spring-summer population.

Akaike’s information criterion (AIC) values for the different models run for the adjusted spring-summer population. The lowest AIC for each pollutant that led to the model selection is highlighted.
